# The neglected burden of snakebites in Cameroon: a review of the epidemiology, management and public health challenges

**DOI:** 10.1186/s13104-017-2718-2

**Published:** 2017-08-14

**Authors:** Joel Noutakdie Tochie, Mazou N. Temgoua, Tsi Njim, Danwang Celestin, Ronni Tankeu, Njinkeng J. Nkemngu

**Affiliations:** 1 Department of Surgery and Sub-Specialties, Faculty of Medicine and Biomedical Sciences, University of Yaoundé I, Yaoundé, Cameroon; 2 Health and Human Development (2HD) Research Group, Douala, Littoral Region Cameroon; 3 Department of Medicine and Specialties, Faculty of Medicine and Biomedical Sciences, University of Yaoundé I, Yaoundé, Cameroon; 4 Centre for Global Health and Tropical Medicine, Nuffield Department of Medicine, University of Oxford, Oxfordshire, UK; 5 Department of Anesthesia, University of Toronto, Toronto, Canada

**Keywords:** Snakebite, Envenomation, Public health challenges, Anti-venom serum, Cameroon

## Abstract

**Objectives:**

Snakebite is an underestimated medical and surgical emergency in developing countries responsible for a high disease burden. Optimal management of snake envenomation in these resource-limited settings is precluded by several public health challenges. In this review, we discuss the disease burden of snakebites in Cameroon and the public health challenges of its management in view of making recommendations essential for policy-making. MEDLINE, African Journals Online and Google Scholar were searched from January 1990 to February 2017 for studies addressing snakebite in Cameroon. Our search extended to include grey literature from book chapters, conference proceedings, theses and documents from organizations.

**Results:**

Our results suggest that snakebites pose a significant health and economic burden in Cameroon. A composite of factors contributes to the challenge of managing snakebites in Cameroon and include: inadequate disease surveillance; poor health-seeking behaviours of patients; under-production and scarcity of anti-venom serum and the relatively high cost of anti-venom serum. There is an urgent need to revamp the current health policies through health education, promotion and building of sustainable health systems. Disease surveillance and management can be improved by providing refresher courses for healthcare providers and subsidization of the prices of anti-venom serum in pharmacies in the country.

## Introduction

Snakebites represent a global public health problem affecting 1.2 to 5.5 million people worldwide each year [1]. Amongst these victims of snakebite, about 421,000 to 1,841,000 true envenomations occur annually, while it is estimated that 20,000–94,000 patients will have a fatal outcome each year [1]. Survivals may sustain venom-induced limb necrosis or gangrene with resultant limb amputation leading to permanent physical disabilities and psychological sequelae [1–3]. The burden of snake envenoming is particularly worrisome in sub-Saharan Africa (SSA) due to preference of traditional remedies as first-line treatment by snake-bitten patients, delayed hospital presentation of snake-bitten patients, scarcity of anti-venom serum (AVS) and financial constraints of patients [4].

Information on the number of snakebites, envenomations, deaths and the frequency of long-term sequelae due to snakebites are essential for evaluating the magnitude of the problem, formulating therapeutic guidelines, planning adequate allocation of resources (particularly AVS) and training health care providers to treat snakebites [1, 2, 4]. However, under-reporting of the incidence of snakebites in rural areas of SSA where snakebites are common has led to underestimation of the true burden of snakebites [1, 4]. Similarly, in Cameroon, evidence has it that snakebites victims are often young, poor farmers and that there is scarcity of AVS all over the national territory [5, 6]. The true disease burden of snakebite has been less well examined in Cameroon and little attention has been attributed to it by health authorities, thus relegating snakebites to a group of neglected public health problems [1–3]. This narrative review synthesizes the current epidemiological data and therapeutic options (particularly AVS) put in place for the management of this pathology in Cameroon, with the goal of providing evidence on the current burden of snakebite in this vulnerable population. This should inform public health authorities on the various public health challenges and the necessary control interventions which can be tailored to curb this burden.

## Main text

### Methods

**Fig. 1:**
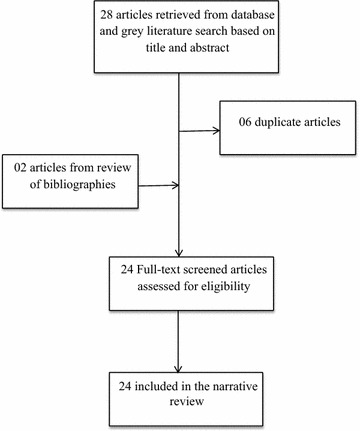
Flow diagram of study selection

We searched three main electronic databases: MEDLINE (via Pubmed), peer-reviewed African Journals Online and Google Scholar from January 1, 1990 to February 28, 2017 for observational studies and randomized controlled trials addressing snakebites in Cameroon. Boolean operators were used with the following key words written in both official languages (English and French) of Cameroon: “snakebite”, “snake envenomation”, “snake envenoming” “anti-venom serum” and “anti-snake venom” cross-referenced with the word “Cameroon” to obtain the maximum possible number of studies addressing the burden of snakebite (prevalence, incidence, morbidity, and mortality) and therapeutic approaches with a focus on treatment using AVS (efficacy, manufacture and distribution) in Cameroon. We scanned the reference lists of articles yielded by the electronic search in order to retrieve additional relevant studies. Our search extended to include grey literature from book chapters, conference proceedings, theses from libraries of medical schools in Cameroon, and statistics from the Ministry of Public Health of Cameron. Eligible articles and grey literature were scrutinised using the modified tool described by Hoy et al. [7] (Table 1). Using data retrieved from a myriad of epidemiological and interventional studies, we sought to provide a synthesis of the most up-to-date and key literature regarding snakebite in Cameroon (Fig. 1).

**Table 1:** Risk of bias assessment tool. Adapted from the risk of bias tool for prevalence studies developed by Hoy et al. [7]

Risk of bias item	Response: yes (low risk) or no (high risk)
*External validity*	
1. Was the study target population a close representation of the national population in relation to relevant variables?	
2. Was the sampling frame a true or close representation of the target population?	
3. Was some form of random selection used to select the sample, OR, was a census undertaken?	
4. Was the likelihood of non-participation bias minimal?	
*Internal validity*	
5. Were data collected directly from the participants (as opposed to medical records)?	
6. Were acceptable case definitions of snakebite used?	
7. Were reliable and accepted diagnostic methods for snake envenomation utilised?	
8. Was the same mode of data collection used for all participants?	
9. Was the length of the shortest prevalence period for the parameter of interest appropriate?	
10. Were the numerator(s) and denominator(s) for the calculation of the prevalence of snakebite appropriate?	
11. Summary item on the overall risk of study bias	
*Low risk of bias* 8 or more ‘yes’ answers	
Further research is very unlikely to change our confidence in the estimate	
*Moderate risk of bias* 6 to 7 ‘yes’ answers. Further research is likely to have an important impact on our confidence in the estimate and may change the estimate	
*High risk of bias* 5 or fewer ‘yes’ answers	
Further research is very likely to have an important impact on our confidence in the estimate and is likely to change the estimate

## Results and discussion

### Epidemiology of snakebite in Cameroon

In Cameroon, snakebites are mainly a rural health problem affecting young farmers aged between 15 and 44 years and the majority of snakebite incidents occur during agricultural activities [5]. Furthermore, the female to male ratio of snake-bitten victims is 1:1.4–1.7 [5, 8], making snakebite an occupational disease and raising considerable concern for the economic impact of the disabilities that may arise in these productive members of society. All 10 regions of the country lack a surveillance system to measure the incidence of snakebites at population-based levels, leading to difficulties in estimations of the exact burden of snakebites in Cameroon [5, 9]. The Northern region of the country is however reported to bear the highest burden, with an average annual incidence of 200 snakebites per 100,000 inhabitants [5]. Nationwide statistics show that Cameroon harbours about 150 snake species, out of which 32 are venomous [6]. The snake species responsible for most of the morbidity and mortality of snakebites in Cameroon are *Echis occellatus* in Sahelian regions [5, 6, 8], *Causus maculatus* in the forest [6] and savannah regions and *Naja melaoleuca* in the forest regions [6].

Though Latin America and SSA account each for about 20% of the global incidence of snakebite, snakebite-related mortality is relatively lower in Latin America when compared to SSA—500 vs. 3500 annual deaths respectively [1]. This discrepancy in mortality may stem from better appraisal of the health problem by the health care system with resultant better snakebite management programs, including the development of locally effective AVS in Latin American countries [1]; components that are not adequate in Cameroon. Further, it is estimated that 50-90% of snake-bitten victims in SSA seek a traditional healer for first-line treatment [4]. This often leads to delayed hospital presentation, a classic predictor of venom-induced consumption coagulopathy [10, 11] and snake envenomation-related mortality [12].

The few available reports from north Cameroon show that venomous snakes are responsible for the following common clinical features of envenomation; oedema (77%), coagulopathy (63%) and necrosis (5%) [5]. In this high-risk zone for snakebites, the mean in-hospital mortality rate is 1% in patients treated with AVS compared to a 5% in hospital settings where AVS is not available [5].

### Public health challenges of snakebites in Cameroon

#### Management of snakebite in Cameroon

At present date, anti-venom immunotherapy is the only specific treatment for snake envenomation [1, 13]. Evidence from local clinical trials has confirmed the safety and efficacy of these sera in the neutralization of snake venom, reversal of acute venom-induced inflammation and haemorrhagic syndrome [14–17]. In general, AVS should be administered as a matter of urgency in the presence of signs of envenomation [18, 19]. Though there is no national guideline on the treatment of snake envenomation in Cameroon, the treatment protocol advocated by some authors entails administration of two 10 ml ampoules of AVS either as direct intravenous injection over five minutes [16, 17] or as an infusion (two 10 ml vials of AVS in 250 ml of normal saline or dextrose solution) over 30 to 60 min [18]. The frequency of re-injections or re-infusions is guided by patient’s clinical conditions [17].

Besides AVS administration, the treatment of snake envenomation involves a number of first aids and adjuvant interventions. A national survey conducted in 2015 confirmed that the majority of health personnel, traditional healers, and snake bitten victims are not conversant with proper first aids measures of snakebites [9]. The most important first aid measures following a snakebite are; rapid wound dressing, immobilization of the bitten limb and non-aggressive analgesia [18, 20]. Previously recommended first-aid measures such as the application of tight ligatures, incision, and suction of the venom are currently condemned by snakebite experts due to the increase of potential adverse effects and the lack of effectiveness [21, 22]. Likewise, traditional treatments involving scarification and application of traditional balms on snakebite wounds may be sources of infections [4]. Adjuvant interventions entail the administration of crystalloids and colloids for hemodynamic instability, anti-fibrinolytics and transfusion of fresh frozen plasma for venom-induced consumption coagulopathy, antibiotics for super-imposed wound infection, anti-tetanus serum for tetanus prevention, mechanical ventilation for respiratory distress and hemodialysis for acute kidney injury [18, 23]. However, the use of this ancillary treatment in most SSA countries is precluded by limited health infrastructures, absence of the necessary drugs and financial constraints of patients [4], as is the case with Cameroon. Noteworthy is a case report of fatal envenomation in a physician bitten by a cobra in an enclaved area of north Cameroon [24]. Despite administration of AVS, his clinical condition rapidly progressed to systemic envenomation with severe respiratory distress warranting urgent endotracheal intubation and mechanical ventilation. The lack of health facilities with these means ultimately led to the death of this physician [24].

#### Anti-venom serum: manufacture and distribution in Cameroon

Two types of polyvalent AVS are available in Cameroon; FAV-Afrique^®^ (Pasteur Merieux Connaught, Lyon, France) and the Serum Institute of India polyvalent anti-snake venom, Sii^®^ supplied by the Cameroon Pasteur Centre and the National Centre for the Supply of Essential Medicines (CENAME) respectively [25]. However, the availability of these two anti-venom sera in all 10 regions of Cameroon remains a serious concern [25]. The number of AVS vials sold annually per million inhabitants has tremendously reduced from 1500 vials in the 1960s to 250 vials in the 1980s and then, less than 50 vials from the year 2000 [5]. This is a serious concern as up to 200 snakebites per 100,000 people annually occur in some regions of Cameroon [5]. Consequently, majority of hospitals’ and private pharmacies in Cameroon, including those located in high-risk zones (Northern Cameroon) for snakebites no longer purchase AVS, do not dispose of a stock of AVS or have just a few vials of AVS [9, 25]. Whereas, the management of severe snake envenomation in Cameroon has previously been shown to require an average of five AVS vials [17]. Several reasons explain the lack of AVS stocks in Cameroon. The first are economic constraints which have compelled the privatisation or withdrawal of several pharmaceutical laboratories involved in the manufacture of AVS for Africa [2]. AVS manufacturers are often vulnerable to uncertainties in market demand and lack of financial investment to upgrade their infrastructure to comply with good manufacturing practices [2, 3]. The second motive is the current ill-defined marketing system of AVS described by Chippaux et al. [5] more than a decade ago. The third reason is the relatively expensive prices of AVS (67–75 U.S. dollars for FAV-Afrique^®^ and 20–23 U.S. dollars for Sii^®^) [25]; prices which are expensive for the average Cameroonian peasant family considering that 9.6% of the population live below the international poverty line [26]. Other factors contributing to the limited stock of AVS in Cameroon are the slow sales of AVS by pharmacies which may lead to financial losses and the lack of means to conserve AVS in rural settings [5, 25]. AVS is not present in the emergency kit of most health facilities [9].

## Conclusion and recommendations

Snake envenomation has a high disease burden in Cameroon, but the extent of the problem is not understood due to poor surveillance systems to adequately record the scope of the problem. This is further compounded by poor health-seeking behaviours, poor infrastructures and the current expensive management options available. The problems associated with this pathology can be resolved if the following recommendations are put in place. A more robust surveillance system should be created with the institution of clear case definitions in health facilities around the country. This should be accompanied by passive surveillance and mandatory reporting into a registry system to establish the scope of the problem in Cameroon and provide data on the incidence, morbidity, and mortality of this neglected health problem. Furthermore, to decrease the prevalence of patients reporting to the hospital with late presentations of this condition, disease prevention, and health promotion activities in communities through health education should be carried out. Also, there is the need for building stronger health systems with specialist services like Intensive Care Units to manage such emergencies. The government should also ensure the availability of anti-venom serum in all health centres and pharmacies in the country at affordable subsidized prices for such vulnerable patients. Refresher courses for health care providers should be carried out on the management of this pathology. These policies will go a long way to build sustainable health systems that could help reduce the burden associated with this condition. Also, the wearing of thick long boots and gloves by farmers as a preventive measure cannot be overemphasized. Finally, the “one health” concept which is absent in Cameroon needs to be instituted. The ministry of health should work in collaboration with other ministries like the ministry of wildlife to identify the various types of snakes in the country and to aid in the development of specific anti-venom serum to target these species.

## Limitations

The main limitation to this study is the heterogeneity across all the studies included, which hindered a meta-analysis.

## Abbreviations

AVS: anti-venom serum
SSA: sub-Saharan Africa
